# A precise berry counting method for in-cluster grapes to guide berry thinning

**DOI:** 10.3389/fpls.2025.1739688

**Published:** 2026-01-09

**Authors:** Wensheng Du, Weishuai Qin, Xiao Cui, Yanjun Zhu, Yonghao Jia, Ruihan Wang, Yuanpeng Du

**Affiliations:** 1 College of Mechanical Engineering, Taishan University, Tai’an, China; 2 School of Biology and Brewing Engineering, Taishan University, Tai’an, China; 3 Business School, Taishan University, Tai’an, China; 4 State Key Laboratory of Crop Biology, Collaborative Innovation Center of Fruit & Vegetable Quality and Efficient Production in Shandong, College of Horticulture Science and Engineering, Shandong Agricultural University, Tai’an, China

**Keywords:** berry thinning, density map, dual-branch network, in-cluster berry counting, MVDNet

## Abstract

In table grape production, berry thinning is a vital management practice where workers remove berries to achieve a target number per cluster. However, this process fundamentally depends on obtaining an accurate initial berry count, which currently relies on manual methods. These conventional approaches are labor-intensive, slow, and error-prone, posing a significant bottleneck to efficient and precise vineyard management. This study proposes a method comprising a dual-branch network named MVDNet and a post-processing algorithm. MVDNet simultaneously performs density map regression for berry counting and bunch segmentation. Its architecture employs a Front-end containing UIB modules for feature extraction, multi-scale feature fusion for spatial detail reconstruction, and a parameter-free SimAM attention mechanism to enhance salient berry features. Extensive experiments demonstrate that our method achieves competitive performance, with MVDNet attaining a Mean Absolute Error (MAE) of 7.7, a Root Mean Square Error (RMSE) of 12.6, and a Mean Intersection Over Union (MIoU) of 0.90 on the test set. Remarkably, our model delivers this high accuracy with extremely low computational resource consumption, containing only 3.372 million parameters, underscoring its suitability for deployment on resource-constrained edge devices. Furthermore, the subsequent post-processing algorithm for per-cluster berry counting achieves a high coefficient of determination (*R*²) of 0.886. The proposed solution thus provides a robust, efficient, and practical tool for automated berry counting, facilitating precise vineyard management and contributing to enhanced grape quality and productivity.

## 1 Introduction

In viticulture, berry thinning is a critical management practice for enhancing berry quality and improving cluster architecture. By selectively removing a portion of the berry set, growers can reduce competition for photosynthetic products and mineral nutrients. Simultaneously, by improving airflow, the risk of fungal diseases is reduced. Therefore, berry thinning is widely regarded as an indispensable technical measure for producing high-quality grapes suitable for winemaking and fresh consumption. The efficacy of thinning operations, however, heavily depends on precision in the removal process. A key aspect lies in the quantitative assessment of berry load before thinning. Accurate berry counting per cluster or vine is essential for achieving target yield parameters and ensuring consistent quality across vineyard blocks. Traditional manual counting methods are not only labor-intensive and time-consuming but are also prone to human error and subjective variability, which can compromise the reliability of thinning outcomes and subsequent yield predictions.

Computer vision has become a key technology for addressing challenges in viticulture. It provides a rapid, non-destructive, and precise means for tasks such as berry counting, which directly improves yield estimation, thinning operations, and data-driven management. Its applications are broad, encompassing visual quality classification (Xiao et al., 2019; Xu et al., 2021; Ye et al., 2022), pest and disease detection (Math and Dharwadkar, 2022; Radhey et al., 2024), and harvesting (Arab et al., 2021; Laurent et al., 2021; Palacios et al., 2023), all contributing to increased efficiency and accuracy in grape production. Focusing on the task of berry counting, methods are broadly categorized into berry-level and cluster-level. Berry-level counting, which aims to identify individual berries across an image for accurate yield prediction, leverages a variety of techniques. These range from traditional methods like morphological operations (Aquino et al., 2018; Luo et al., 2021) and contour analysis (Liu et al., 2020) to modern deep learning approaches such as object detection and semantic segmentation (Buayai et al., 2021; Zabawa et al., 2020; Du and Liu, 2023), all of which have demonstrated high accuracy.

In contrast, cluster-level counting has been less studied and presents greater technical challenges. This task requires not only identifying and counting the berries in an image but also accurately determining the total number of berries contained within each grape cluster. Woo et al. (2023) employed YOLOv5 for detecting grape clusters and visible berries, combined with a random forest regressor to estimate total berry count, achieving real-time performance on mobile devices with an MAE of 2.6. While this approach demonstrates notable efficiency, its reliance on handcrafted features from bounding boxes may affect adaptability under more challenging scenarios, such as significant occlusion or high inter-berry morphological variation. Yang et al. (2024) developed a Probability Map-based Grape Detection and Counting (PMGDC) framework using U-Net to generate probability maps for both clusters and berries. Their method incorporates three post-processing algorithms to facilitate cluster localization, berry counting, and berry-per-cluster estimation, thus enhancing functional integration. Nevertheless, the framework involves computationally intensive post-processing steps and may encounter difficulties in accurately distinguishing overlapping clusters. Further advancing this direction, Yang et al. (2025) proposed Mask-GK, which encodes instance-level annotations into structured probabilistic representations via mask-based Gaussian kernels. By applying watershed segmentation to these probability maps, their method jointly addresses berry segmentation and counting, reporting an MAE of 9.32 and an AP of 0.665 on the public GBISC dataset. Despite these promising results, the approach relies on detailed annotations that can be resource-intensive, and segmentation performance may be constrained with larger or non-contiguous berry instances.

In summary, prevailing techniques remain constrained in feature representation, occlusion handling, and system-level integration. To overcome these limitations, a dual-branch network and a post-processing algorithm are proposed. Through a unified learning strategy, our network achieves high-precision berry counting and accurate cluster segmentation without relying on manual feature extraction, while also simplifying post-processing. The presented method enhances robustness in berry counting, offering a reliable and efficient visual intelligence solution for precision viticulture. The contributions of this work are as follows:

1. MVDNet, a dual-branch network architecture that synergistically integrates berry density map regression and bunch segmentation, is proposed. The proposed model comprises three key components: a UIB-based Front-end, a multi-scale feature fusion module, and a parameter-free SimAM attention mechanism. This integrated design significantly enhances feature extraction, leading to high accuracy in grape berry counting and cluster segmentation.
2. Experimental results confirm that MVDNet achieves high computational efficiency and accuracy. With only 3.372M parameters, it attains an MAE of 7.7, RMSE of 12.6, and MIoU of 0.90 on the test set. This performance demonstrates its superiority over comparable models and suitability for resource-constrained edge devices.
3. A robust, image processing-based post-processing algorithm is developed to translate the MVDNet outputs into accurate per-bunch berry counts. This method achieves a high *R*² of 0.886 on the test set, providing a practical and automated solution to guide berry thinning operations for precise yield management and enhanced grape quality.

## 2 Materials and methods

### 2.1 Dataset of table grapes at the fruit set stage

A dataset of table grape berries was constructed to train and test the method proposed in this research. The images of table grapes were taken from 8:00 to 15:00 each day, over a total of three days (all sunny days) from May 28th to May 30th, 2021, at the horticultural experiment station of Shandong Agricultural University, Daiyue District, Tai’an City, Shandong Province, China (longitude 117° 9’ 38.92” E, latitude 36° 9’ 47.4” N). The ‘Shine Muscat’ table grapes were trained in an overhead trellis system. The ‘Shine Muscat’ grape has undergone seedless treatment. The shooting device was an iPhone SE mobile phone with a resolution of 3,024 x 4,032.

**Figure 1:**
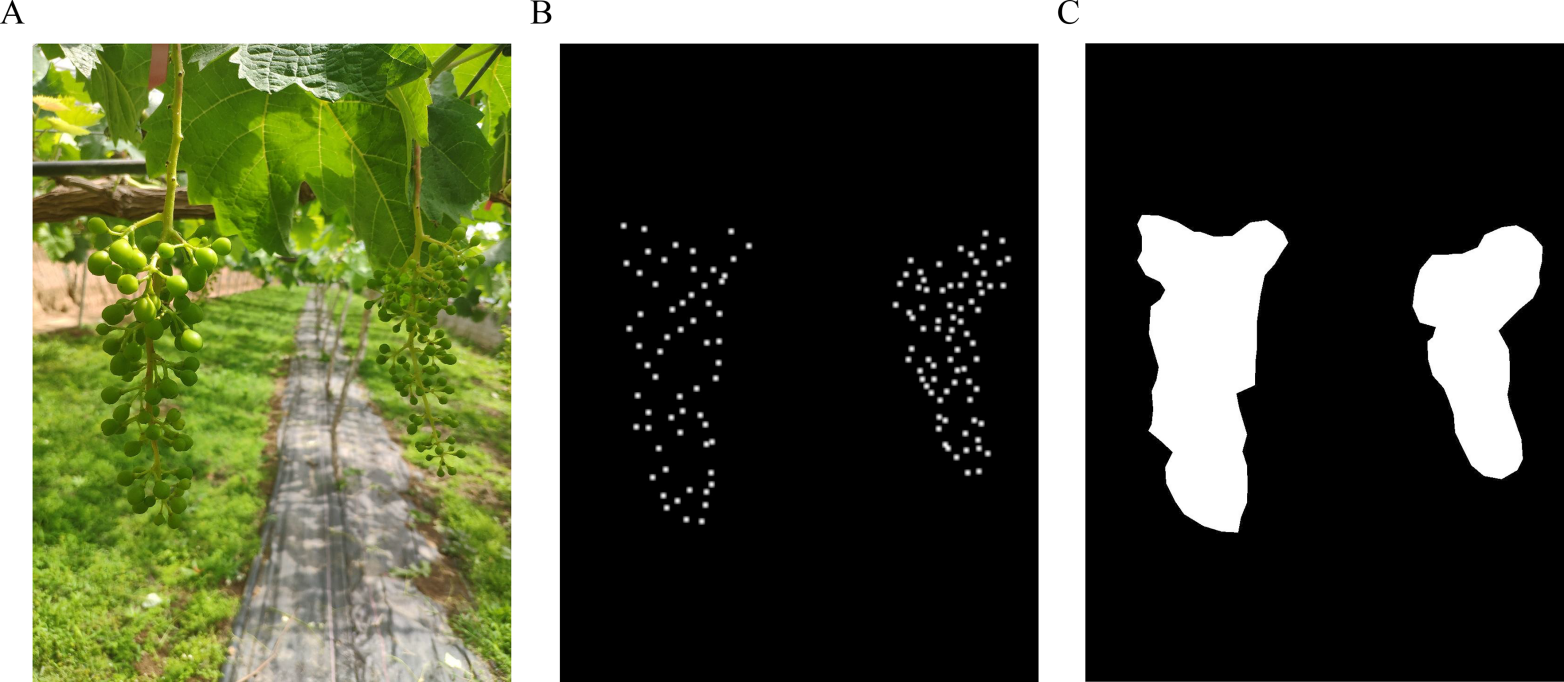
Labelled images. **(A)** Original image, **(B)** Density map, and **(C)** Mask.

Images of table grapes during the berry thinning period were collected in a natural environment, and a total of 779 images were manually screened, of which 545 were in the training set, 155 were in the validation set, and 79 were in the test set. The distribution of the dataset is shown in **Table 1**. The captured images were scaled to 1,024 x 1,365 to reduce the loading and computation time of the images. Using the specialized annotation tool Labelme, grape berries were annotated as points, while grape bunches were delineated with polygons. The masks of grape bunch were generated by preprocessing as shown in **Figure 1C**. To reduce storage burden and accelerate the training process, density maps corresponding to grape berries, as shown in **Figure 1B**, were generated in an online manner. Point maps were produced by processing the annotation files, with each point map comprising a set of locations corresponding to individual grape berries in the image. For a given image containing *N* grape berries, each berry positioned at pixel *x_i_* can be formally represented as:

**Table 1:** The distribution of the dataset.

Classification of datasets	Number of images	Number of berries	Number of clusters
Training dataset	545	49960	1102
Validation dataset	155	14650	315
Test dataset	79	7671	132

(1) $$H(x)=\sum_{i=1}^{N}\delta (x-x_{i})$$

The function *H*(*x*) in Equation 1 is convolved with a Gaussian kernel $G_{\sigma }(x)$ to obtain a continuous density function as shown in Equation 2.

(2) $$F(x)=H(x)•G_{\sigma }(x)=\sum_{i=1}^{N}\delta (x-x_{i})•G_{\sigma }(x)$$

### 2.2 Overview of the method

**Figure 2:**
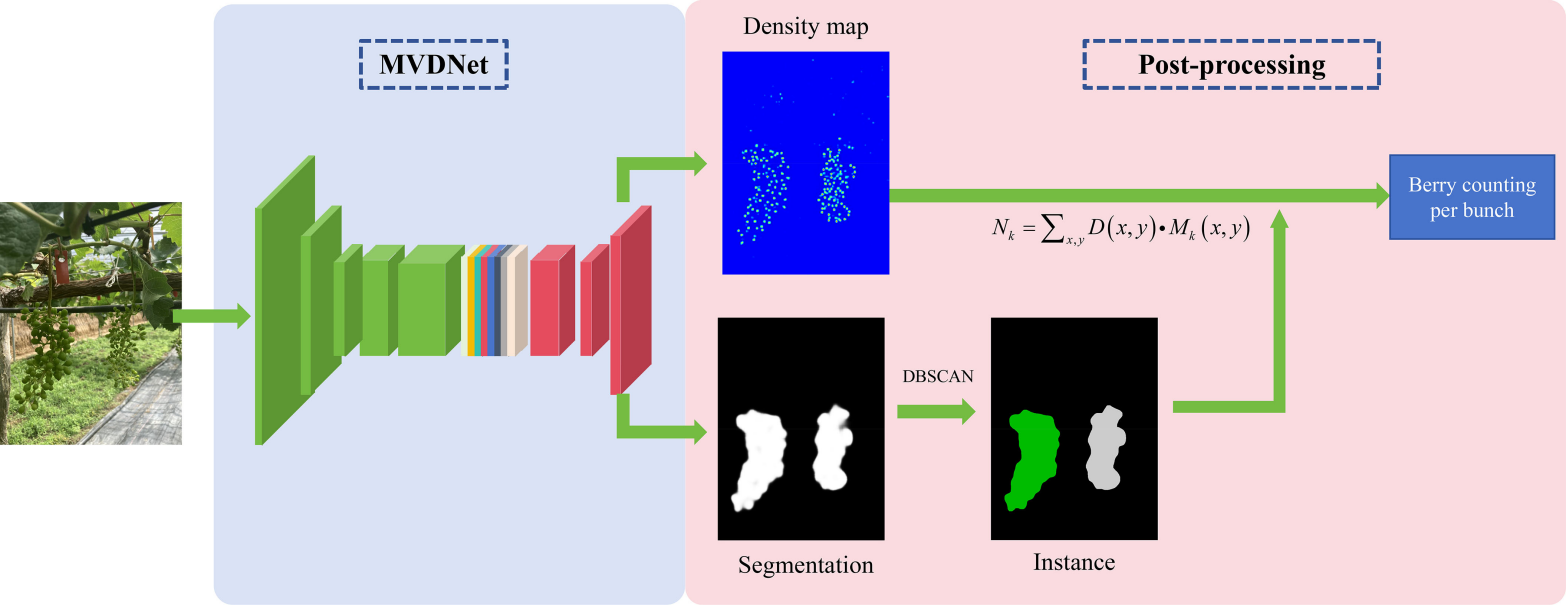
The method of berry counting per bunch.

The presented methodology comprises two core components: a dual-branch network and a post-processing algorithm as shown in **Figure 2**. First, to address the challenges posed by small berry size, significant occlusion, and complex environmental interference during the berry thinning period, a lightweight network named MVDNet was designed, which balances high accuracy and dual-task performance. This network simultaneously performs berry density map regression and grape cluster segmentation. The input images are processed through the dual-branch multi-task network to generate a density map of grape berries and a preliminary segmentation mask of the grape clusters. Subsequently, a post-processing algorithm refines the segmentation mask to achieve instance-level separation and precise localization of individual grape clusters. Within each segmented cluster region, the number of berries is calculated by integrating the density map, enabling accurate berry counting per cluster. This method can be applied to vineyards in complex environments, providing a reliable and efficient technical solution for automated berry thinning.

### 2.3 Structure of MVDNet

**Figure 3:**
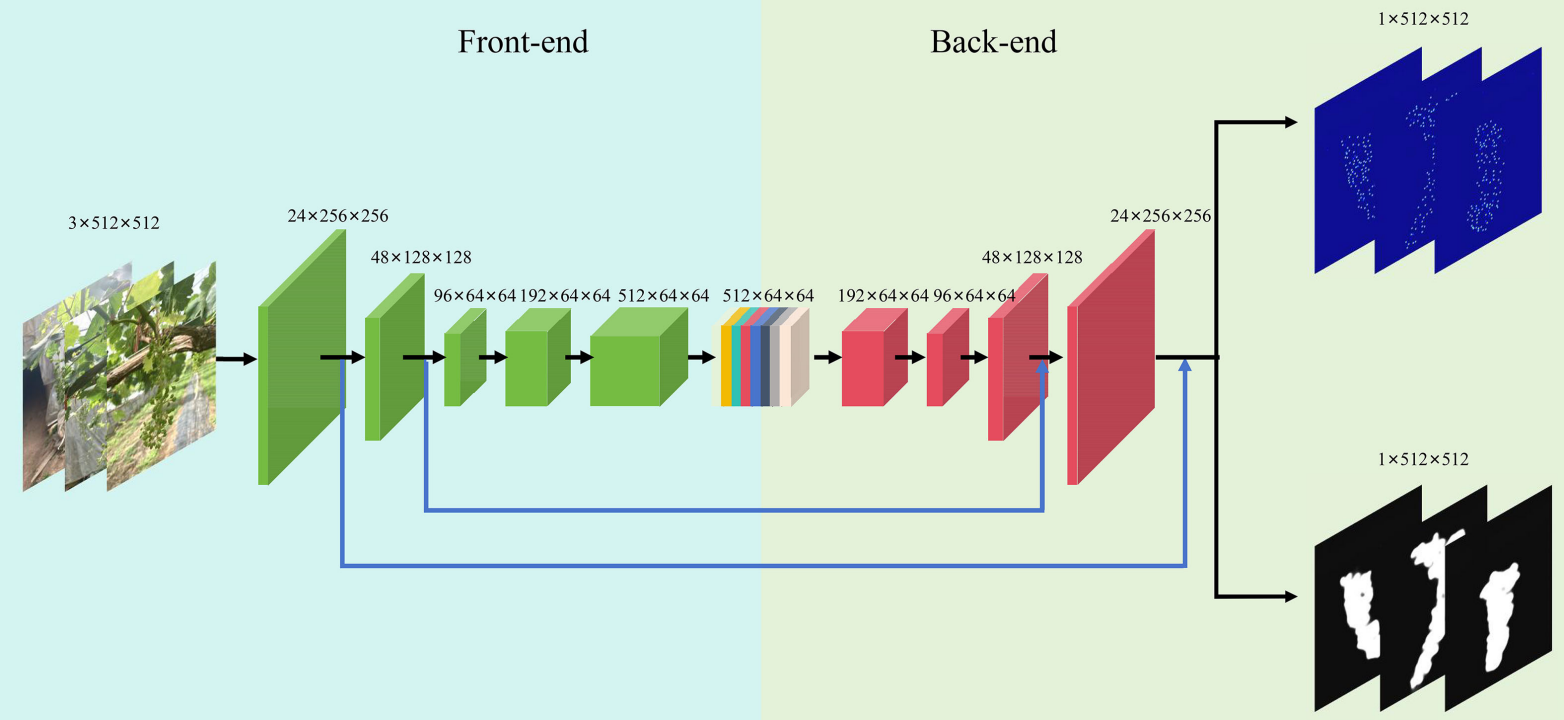
Structure of MVDNet.

Our basic idea is to design a lightweight network that achieves high predictive accuracy while maintaining low computational complexity. The proposed model adopts a dual-branch encoder-decoder architecture as shown in **Figure 3**, structurally inspired by U-Net (Ronneberger et al., 2015), which enables simultaneous grape berry density map regression and grape cluster segmentation. In the Front-end, a shared encoder performs three-stage downsampling to progressively extract multi-scale features from the input image. To enhance feature representation, a feature fusion mechanism is incorporated to adapt to berries of varying sizes, while a lightweight attention module is integrated to strengthen the model’s ability to capture discriminative local characteristics of berries.

The Back-end consists of a symmetric three-stage upsampling pathway, where skip connections are utilized to incorporate fine-grained details from the encoder, thereby gradually restoring spatial information. Based on this shared network, two task-specific branches are derived in the decoding path: a regression branch dedicated to reconstructing high-precision berry density maps for localization and counting of individual berries, and a segmentation branch that aggregates multi-scale contextual semantic information to accurately delineate cluster boundaries, effectively separating them from the background and adjacent clusters. This synergistic dual-branch design not only enables microscopic analysis at the berry level through density estimation, but also supports macroscopic examination at the cluster level via pixel-wise segmentation, thereby providing a comprehensive and efficient solution for in-cluster berry counting.

#### 2.3.1 Structure of front-end

**Figure 4:**
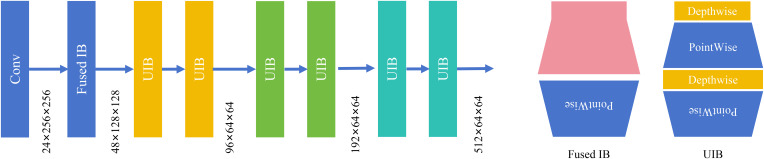
Structure of front-end.

The Front-end is constructed as a hierarchical feature pyramid for progressive spatial compression and channel expansion, as shown in **Figure 4**. The process initiates with a standard convolutional layer, producing a feature map with 24 channels at a 256×256 resolution. This is immediately followed by a Fused Inverted Bottleneck (Fused IB) module, which doubles the channel count to 48 while halving the spatial dimension to 128×128, achieving an initial efficient downsampling. The core of the Front-end then consists of a sequence of four Universal Inverted Bottleneck (UIB) stage, which is proposed in MobileV4 (Qin et al., 2025). The first UIB stage further increases channels to 96 and reduces the resolution to 64×64. The subsequent two UIB stages progressively expand the channel capacity to 192 and 512, respectively, while maintaining the 64×64 resolution, thereby deepening the feature representation without sacrificing spatial granularity. This systematic design ensures a computationally efficient and representatively powerful initial feature extraction. It effectively establishes a rich, multi-scale feature hierarchy that serves as a robust foundation for subsequent network components, balancing the critical trade-offs between model accuracy, parameter efficiency, and computational speed.

#### 2.3.2 SimAM

**Figure 5:**
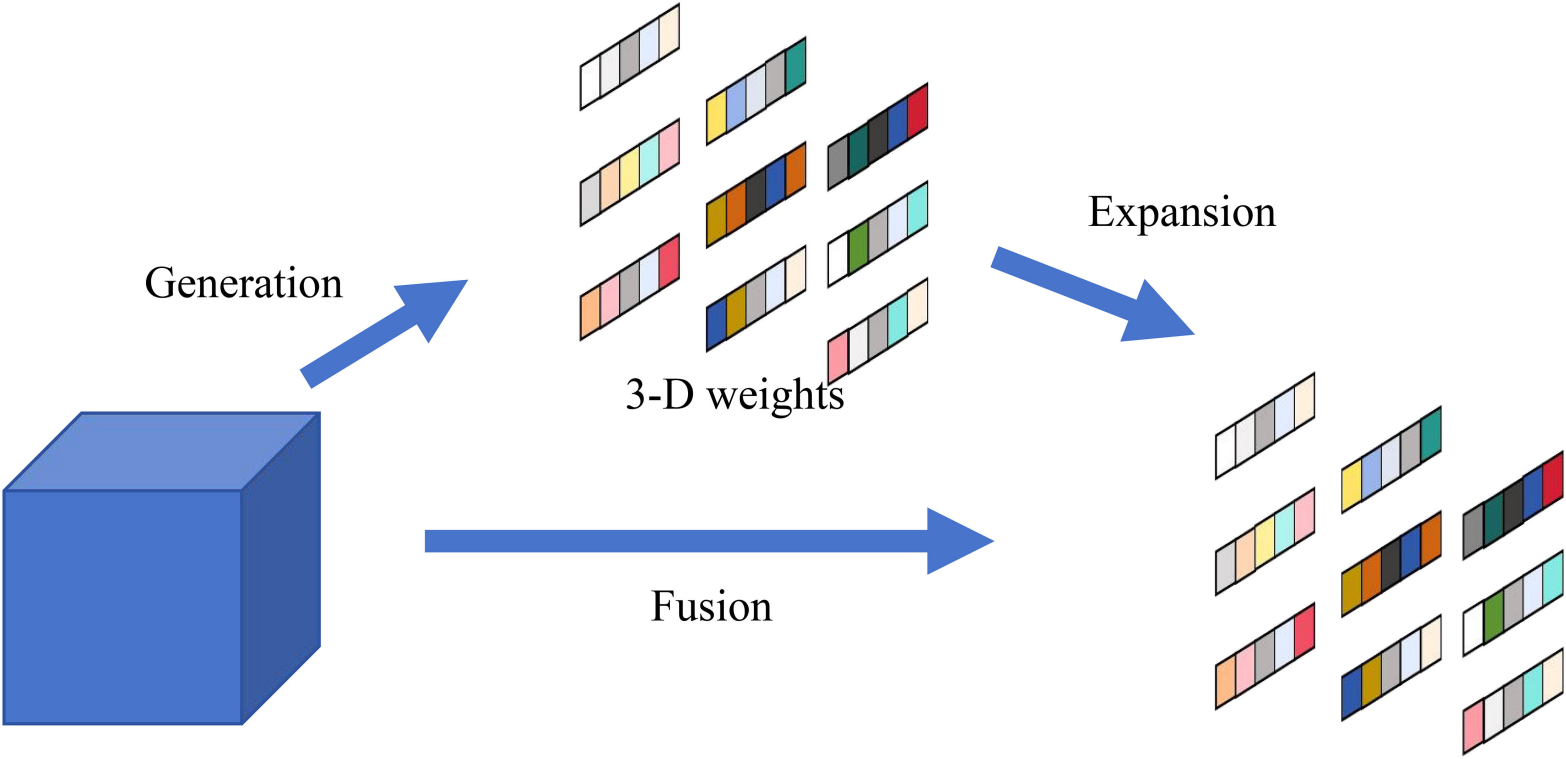
Structure of SimAM.

In the design of dual-branch network architecture, a critical challenge lies in effectively modeling cross-dimensional dependencies without introducing excessive computational complexity. To address this fundamental requirement, the SimAM attention module (Yang et al., 2021) is incorporated. Most existing attention modules generate 1-D or 2-D weights from feature X, and then extend the generated weights to channel and spatial attention. The SimAM module estimates 3D attention weights directly through a parameter-free process centered on an energy function. This function assesses the linear separability of each neuron from its neighbors, assigning higher importance (lower energy) to more distinctive neurons. The resulting energies are normalized and scaled with a Sigmoid function to produce the final 3D attention map, which is then multiplied with the input features to perform adaptive feature refinement. This mechanism, illustrated in **Figure 5**, enhances feature representation without adding any parameters to the model. The energy function *e_t_* corresponding to its neuron is defined as Equation 3:

(3) $$e_{t}\left(w_{t},b_{t},y,x_{i}\right)=\frac{1}{M-1}\sum_{i=1}^{M-1}{\left(-1-\left(w_{t}x_{i}+b_{t}\right)\right)}^{2}+{\left(1-\left(w_{t}t+b_{t}\right)\right)}^{2}+\lambda w_{t}^{2}$$

where *t* and *x_i_* are the target neuron another neurons in the same channel of input feature $X\in {\mathbb{R}}^{C\times H\times W}$. *M* is the neurons number, M=H×W. $w_{t}$ (see Equation 4) and $b_{t}$ (see Equation 5) are the weight and bias of the target neuron, λ is a small value (default 1e-4), serving as a numerical stabilizer, which prevent division-by-zero errors when feature variance approaches zero.

(4) $$w_{t}=-\frac{2(t-\mu_{t})}{{\left(t-\mu_{t}\right)}^{2}+2\sigma_{t}^{2}+2\lambda }$$

(5) $$b_{t}=-\frac{1}{2}(t+\mu_{t})w_{t}$$

where $\mu_{t}=\frac{1}{M}\sum_{i=1}^{M}x_{i}$ and $\sigma_{t}^{2}=\frac{1}{M}{\sum_{i}^{M-1}\left(x_{i}-\mu_{t}\right)}^{2}$ are the mean and variance calculated for all neurons except *t* in the channel.

The minimal energy $e_{t}^{*}$ can be computed as Equation 6:

(6) $$e_{t}^{*}=\frac{4({\hat{\sigma }}^{2}+\lambda )}{{\left(t-\hat{\mu }\right)}^{2}+2{\hat{\sigma }}^{2}+2\lambda }$$

where $\hat{\mu }=\frac{1}{M}\sum_{i=1}^{M}x_{i}$ and ${\hat{\sigma }}^{2}=\frac{1}{\text{M}}{\sum_{i}^{M-1}\left(x_{i}-\hat{\mu }\right)}^{2}$.

(7) $$\tilde{X}=sigmoid\left(\frac{1}{E}\right)⊙X$$

where E groups all $e_{t}^{*}$ values across the channel and spatial dimensions. The sigmoid function constrains excessively large values within E in Equation 7.

#### 2.3.3 Multi-scale feature fusion

**Figure 6:**
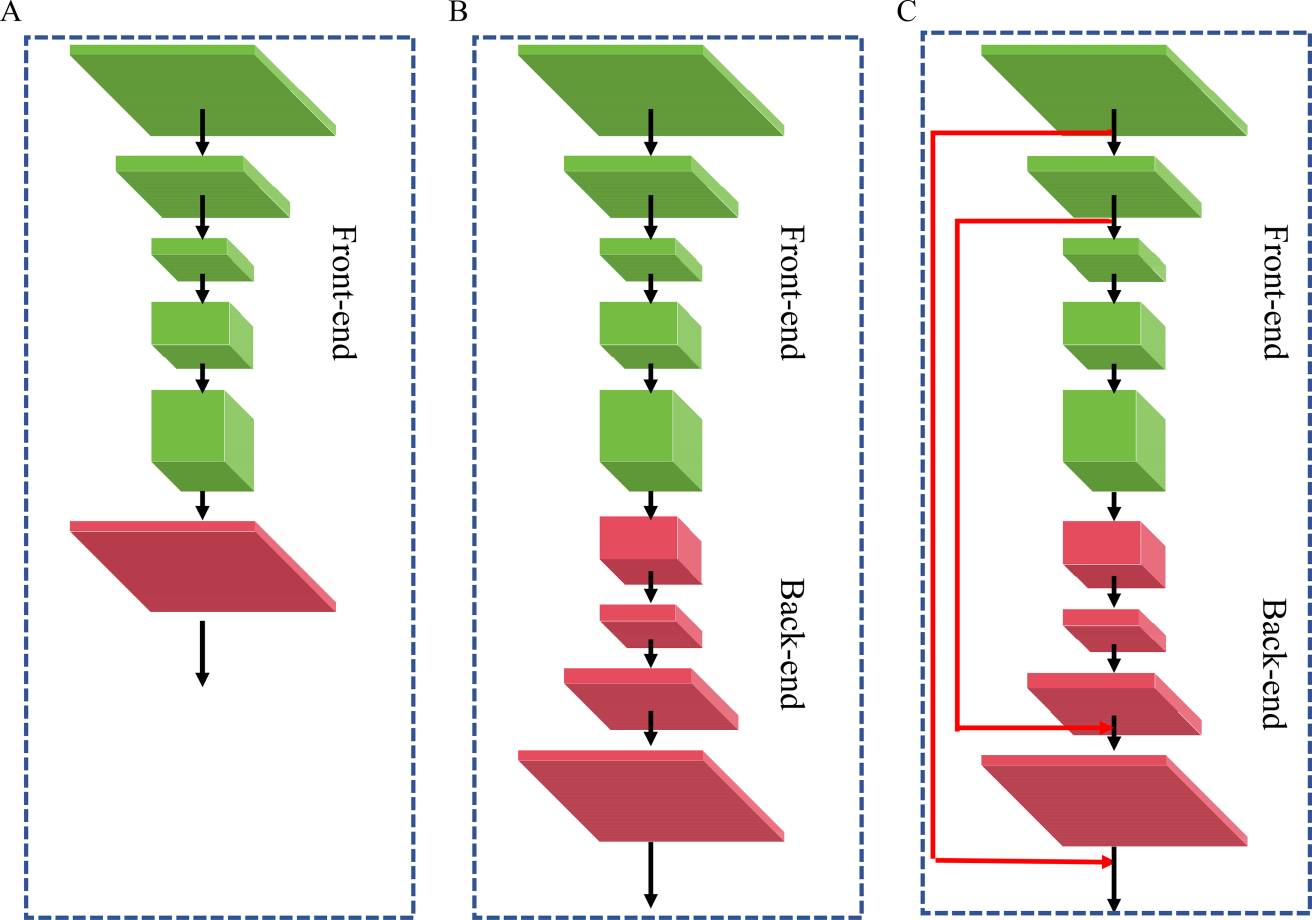
Different network structures. **(A)** Front-end **(B)** Front-end + Back-end **(C)** Front-end + Back-end + Multi-scale feature fusion.

To address the inherent disparities in feature scale and semantic hierarchy within dual-branch architectures, this study employs a multi-scale feature fusion strategy. This design effectively mitigates feature misalignment between branches through cross-level feature interactions, thereby enabling the network to fully leverage the complementary advantages of each branch and significantly enhance detection accuracy and robustness. Three networks are designed and compared in our work, as shown in **Figure 6**. The first network outputs a low-resolution feature map and then generates the density map directly via a simple bilinear interpolation in **Figure 6A**. The second network consists of a series of convolutional layers. It is responsible for decoding the high-level semantic feature maps extracted from the Front-end back to the original input resolution and regressing the density maps in **Figure 6B**. The last network is based on “Front-end + Back-end” and multi-scale feature fusion is adopted to gradually integrate different levels of features in the Back-end up-sampling process in **Figure 6C**.

It significantly enhances model performance through cross-hierarchy feature fusion. On one hand, incorporating shallow high-resolution features directly compensates for detail loss during upsampling, improving boundary precision and reducing background artifacts. On the other hand, this design dynamically aggregates receptive fields across feature hierarchies: enhancing small-target localization while strengthening robustness for large-target clusters, especially under occlusion. Moreover, multi-scale feature fusion provides shortcut paths for gradient flow, alleviating vanishing gradients and accelerating convergence.

#### 2.3.4 Loss of the model

The total loss of this model is the weighted sum of the density map loss $L_{den}$and image segmentation loss $L_{seg}$. The total loss is defined as Equation 8:

(8) $$L_{total}\;=\lambda_{den}\;\cdot L_{den}+\lambda_{seg}\;\cdot L_{seg}$$

where $\lambda_{den}$ and $\lambda_{seg}$ are weights used to balance the relative importance of two tasks. An adaptive weight scheduler is employed to dynamically balance the contributions of two loss functions during training, with initial values of $\lambda_{den}$ and $\lambda_{seg}$ are 1 and 0.01, respectively. This module automatically adjusts the loss weights at regular intervals based on the recent optimization history. The average loss for each task over a fixed window is computed, and new weights are proposed inversely proportional to their ratios relative to the minimum loss. These proposed weights are then integrated via exponential smoothing with a factor to ensure stable transitions, followed by a normalization step to maintain consistent gradient magnitudes. This strategy continuously redistributes the learning focus towards more challenging tasks, promoting robust and balanced multi-task optimization without the need for manual weight tuning.

The density map loss uses the MSE loss function (see Equation 9):

(9) $$L_{den}=\frac{1}{N}{\sum_{i=1}^{N}\left(D_{pred}^{i}-D_{true}^{i}\right)}^{2}$$

where *N* is the number of pixels in a batch of images, $D_{pred}^{i}$ is the *i*-th density value predicted by the model, $D_{true}^{i}$ is the i-th true density value.

To balance pixel-level classification accuracy and overall region shape matching, the image segmentation loss uses binary cross-entropy loss $L_{BCE}$ (see Equation 11) and dice loss $L_{Dice}$ (see Equation 12), which is defined as Equation 10:

(10) $$L_{seg}=\alpha \cdot L_{BCE}+\beta \cdot L_{Dice}$$

where α and β are weights used to balance the relative importance of two losses, the initial values of α and β are both 1.

(11) $$L_{BCE}\;=-\frac{1}{N}\sum_{i=1}^{N}\left[S_{true}^{i}\cdot log(\sigma (S_{pred}^{i\;}))+(1-S_{true}^{i}\;)\cdot log(1-\sigma (S_{pred}^{i}\;))\right]$$

where $S_{pred}^{i\;}$ is the i-th segmentation logits value output of the model, $S_{true}^{i}$ is the real i-th segmentation label (0 or 1), σ is the sigmoid function, $\sigma (x)=\frac{1}{1+e^{-x}}$.

(12) $$L_{Dice\;}=1-\frac{2\sum_{i=1}^{N}\sigma (S_{pred\;}^{i})\cdot S_{true\;}^{i}+\epsilon_{g}}{\sum_{i=1}^{N}\sigma (S_{pred\;}^{i})+\sum_{i=1}^{N}S_{true\;}^{i}+\epsilon_{g}}$$

where $\epsilon_{g}$ is a small smoothing constant used to prevent division by zero.

**Figure 7:**
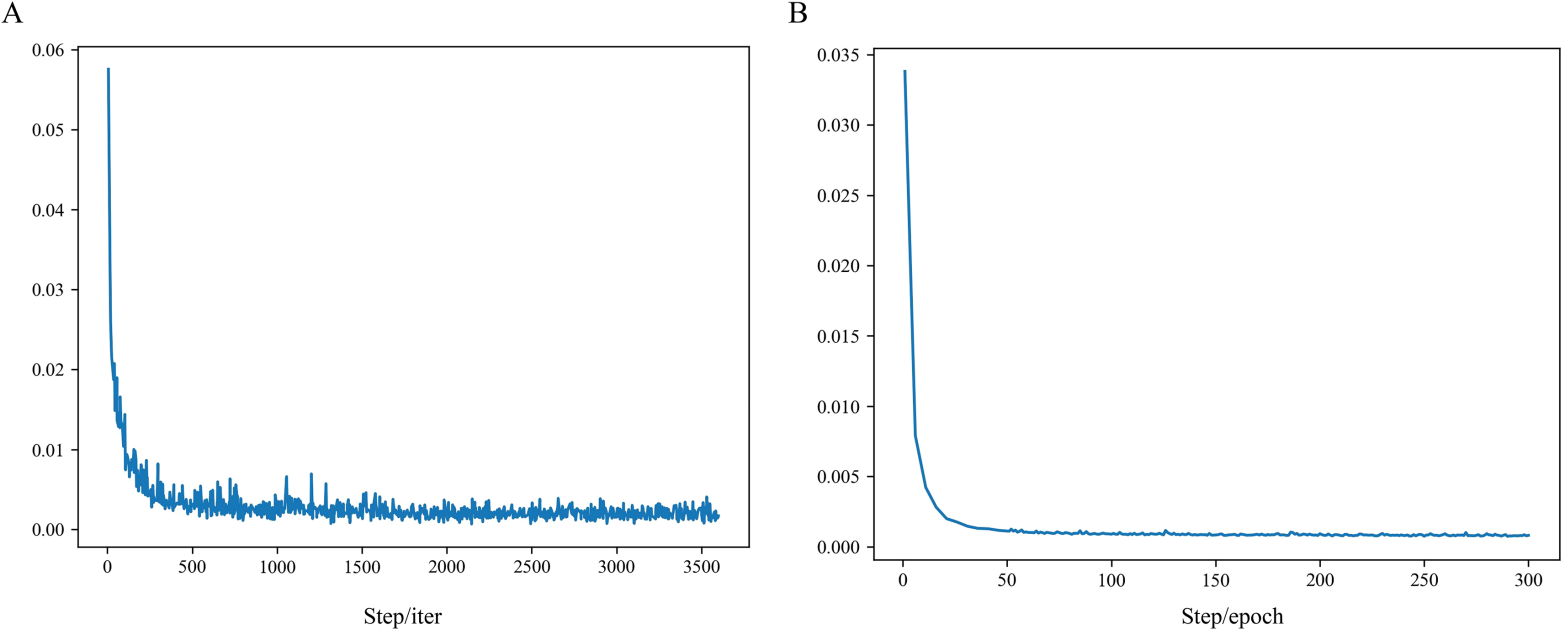
Loss curve. **(A)** Training loss, and **(B)** Validation loss.

The training loss and validation loss of the model are shown in **Figure 7**, and it can be seen that with the gradual increase in the number of epochs or steps, both of them gradually decrease, and no overfitting is produced.

### 2.4 Berry counting per cluster via post-processing

The berry count for each grape bunch is derived through a post-processing pipeline that integrates the outputs of the two branches of our network: the bunch segmentation map S(x,y) and the berry density map D(x,y). The procedure is summarized as follows:

#### 2.4.1 Binarization and morphological processing

The proposed approach first binarizes the input segmentation map S(x,y) through thresholding to obtain the binary map $B(x,y)=\{\begin{array}{c} 1\;if\;S(x,y)>0.5\\ 0\;otherwise \end{array}$. The morphological operations, including the closing operation $B_{closed}=(B⊕K)⊖K$ and the opening operation $B_{cleaned}=(B_{closed}⊖K)⊕K$ are employed to remove noise and fill holes.

#### 2.4.2 Contour extraction and filtration

The contour extraction phase identifies external boundaries through connected component analysis, followed by area-based filtration using the integral formula $A_{i}=\frac{1}{2}|\oint_{C_{i}}\left(xdy-ydx\right)|$ to exclude contours below the minimum area threshold $A_{\min}$.

#### 2.4.3 Centroid calculation

For each retained contour, its centroid $\left(c_{x},c_{y}\right)$ is calculated as a center point, where $c_{x}=\frac{M_{10}}{M_{00}}$ and $c_{y}=\frac{M_{01}}{M_{00}}$, with $M_{pq}$ representing the spatial moments of the binary region $M_{pq}=\sum_{x}\sum_{y}x^{p}y^{q}B(x,y)$.

#### 2.4.4 Center clustering with DBSCAN

These center points are then clustered using the DBSCAN algorithm, which groups spatially proximate points based on Euclidean distance $d(p_{i},p_{j})=\sqrt{{\left(c_{x_{i}}-c_{x_{j}}\right)}^{2}+{\left(c_{y_{i}}-c_{y_{j}}\right)}^{2}}$ to form candidate clusters; the number of clusters is constrained to a maximum of $N_{\max}$, prioritizing those with the largest total area. The DBSCAN sensitivity is primarily controlled by two parameters: neighborhood radius (*ϵ*) and minimum points (minPts).

#### 2.4.5 Berry count estimation

An instance mask is generated for each cluster, and the berry count per grape cluster is estimated by integrating the density map D(x,y) as $N_{k}=\sum_{x,y}D(x,y)\;\cdot \;M_{k}(x,y)$, where $M_{k}(x,y)$represents the binary mask of the *k*-th cluster.

## 3 Experiments and results

### 3.1 Experimental platforms and model training

The model training and prediction in this study were executed on a Graphics Processing Unit (GPU) server. The experimental configurations are detailed in **Table 2**. All models were trained for 300 epochs using a learning rate of 1e-4, Adam optimizer, and a weight decay factor of 1e-4. During training, each image was cropped to 512×512, and each batch is 4. An early stopping criterion was applied with a patience of 50 epochs, halting training if the validation performance failed to exceed the best recorded metric for 50 consecutive epochs.

**Table 2:** Experimental configuration.

Configuration	Parameter
CPU	Intel Core i7
GPU	NVIDIA GeForce RTX 3090 (24GB)
Operation system	Windows 10
CUDA	11.1
Programming language	Python 3.8
Deep Learning Framework	PyTorch 1.9.0
Compiler software	Pycharm

Our model was implemented based on ‘NWPU-Crowd’ (Wang et al., 2021), a large benchmark framework for crowd counting. In this framework, the image preprocessing and the evaluation criteria of the models were the same, which could ensure the fairness of model performance comparison. The task of this framework was migrated from crowd counting to grape berry counting to evaluate the performance of the proposed model. All architectures were restructured into a dual-branch framework to facilitate a comparative analysis between density-based regression of grape berries and segmentation performance of grape clusters.

MAE and RMSE were employed to evaluate the density map regression performance of all models, and MIoU was used to assess the segmentation performance of all models. MAE, RMSE, and MIoU are shown in (Equations 13–15).

(13) $$\mathrm{MAE}=\frac{1}{N}\sum_{i=1}^{N}\left|B_{i}\;-\;B_{i}^{gt}\right|$$

(14) $$\text{RMSE}=\sqrt{\frac{1}{N}{\sum_{i=1}^{N}\left|B_{i}\;-\;B_{i}^{gt}\right|}^{2}}$$

(15) $$\text{MIoU}=\frac{1}{N_{\text{C}}}\sum_{i=1}^{N_{C}}\frac{p_{ii}}{\sum_{j=1}^{N_{C}}P_{ij}+\sum_{j=1}^{k}P_{ji}-P_{ii}}$$

where *N* is the number of test images, *B_i_* is the number of grape berries predicted by the model in each image, $B_{i}^{gt}$is the number of actual grape berries in each image, *N_C_* is the number of classes including the background class, $P_{ii}$ is the number of pixels where the ground truth and predicted values are both *i*, $P_{ij}$ is the number of pixels where the ground truth is *i* but the predicted value is not *j*, $P_{ji}$ is the number of pixels where the ground truth is not *j* but the predicted value is *i*.

### 3.2 Ablation experiment

This study systematically evaluated the impact of different module combinations on grape berry density map regression and grape cluster image segmentation performance, revealing the contributions of each module and its underlying mechanisms. As summarized in **Table 3**, using only the Front-end module yielded baseline results with an MAE of 9.0, RMSE of 13.4, and MIoU of 0.85. The inclusion of the Back-end module reduced MAE by 0.5 and RMSE by 0.2, while improving MIoU by 0.01, indicating enhanced feature representation and contextual integration. Further integration of the Multi-scale Feature Fusion module led to additional reductions in MAE (by 0.3) and RMSE (by 0.3), along with a 0.02 gain in MIoU, attributed to improved multi-scale feature extraction and segmentation consistency. Finally, incorporating the SimAM attention module achieved the best performance: MAE decreased by 0.5, RMSE by 0.3, and MIoU increased by 0.02, resulting in final values of 7.7, 12.6, and 0.90, respectively. This improvement was attributed to SimAM’s ability to adaptively enhance salient spatial and channel-wise features while suppressing irrelevant information.

**Table 3:** Results of the ablation experiment.

Front-end	Back-end	Multi-scale feature fusion	SimAM	MAE	RMSE	MIoU
✓				9.0	13.4	0.85
✓	✓			8.5	13.2	0.86
✓	✓	✓		8.2	12.9	0.88
✓	✓	✓	✓	7.7	12.6	0.90

### 3.3 Impact of different attention mechanisms on modeling

For further validation of the effectiveness of the SimAM module, the effects of different attention mechanism modules [SE (Hu et al., 2020), CA (Hou et al., 2021), ECA (Wang et al., 2020), CBAM (Woo et al., 2018), SA (Zhang and Yang, 2021)] on the model were compared as shown in **Table 4**. It could be seen that the SimAM module demonstrates superior performance, achieving the best results while maintaining a highly competitive parameter count of only 3.372M. Although modules like SA could match SimAM in MIoU (0.89), they exhibit significantly higher MAE and RMSE, indicating less accuracy in density estimation. Conversely, while CA and CBAM achieved a low MAE of 8.5, they fall short of SimAM’s segmentation accuracy (MIoU). Notably, the SE module, despite a high MIoU, performed poorly on the density estimation task, suggesting a limitation in optimizing for regression objectives. The key advantage of SimAM lied in its parameter-free design, which directly estimated the importance of each neuron from a neuroscience perspective without adding extra trainable parameters to the network. This allowed SimAM to more effectively suppress irrelevant background noise and enhance discriminative berry features in complex vineyard scenes, leading to simultaneous improvements in regression accuracy and segmentation fidelity.

**Table 4:** Results of the different attention blocks.

Attention blocks	MAE	RMSE	MIoU	Param/M
SE	9.0	13.4	0.89	3.4
CA	8.5	13.0	0.86	3.395
ECA	8.3	12.7	0.86	3.369
CBAM	8.0	12.8	0.88	3.407
SA	8.0	13.0	0.89	3.369
SimAM	7.7	12.6	0.90	3.372

### 3.4 Comparative experiment

To further validate the performance of the MVDNet model, it was compared with four typical counting models based on density maps, MCNN (Zhang et al., 2016), CSRNet (Li et al., 2018), CANNet (Liu et al., 2019), and SCAR (Gao et al., 2019), and the results were shown in **Table 5**. Among all the models, MVDNet demonstrated superior performance in both grape berry density map regression and grape bunch segmentation compared to other models. For density map regression, MVDNet achieved the lowest errors, significantly outperforming models like CSRNet (MAE: 9.0, RMSE: 15.1) and CANNet (MAE: 9.9, RMSE: 14.9). This indicated a more accurate estimation of the number and distribution of berries. Simultaneously, for the segmentation task, MVDNet attained the highest MIoU of 0.89, surpassing SCAR (MIoU: 0.87) and other counterparts, reflecting its exceptional ability to delineate the precise boundaries of grape bunches.

**Table 5:** Results of different models.

Models	MAE	RMSE	MIoU	Param
MCNN	14.2	21.2	0.73	138.058K
CSRNet	9.3	16.9	0.80	16.282M
CANNet	10.3	14.8	0.80	18.122M
SCAR	9.0	16.8	0.87	16.306M
MVDNet	7.7	12.6	0.90	3.372M

The underlying reason for MVDNet’s outstanding performance, despite having a significantly smaller parameter count than competitors like CSRNet (16.282M) and SCAR (16.306M), lied in its efficient architectural design, which employed a substantial number of UIB modules within the Front-end network. The core UIB module was pivotal, as its lightweight, identity-based structure facilitates efficient gradient flow and feature reuse, which was crucial for learning the complex textures of grape clusters without a significant parameter overhead. Furthermore, the integration of the SimAM enhanced feature discrimination by explicitly modeling the linear separability between features, allowing the network to focus on more informative berry and edge regions while suppressing redundant background information. Finally, the multi-scale feature fusion mechanism effectively aggregated contextual information at various receptive fields, enabling the model to robustly handle the large-scale variations inherent in grape berries within an image. Consequently, MVDNet not only provided more precise density counts by better localizing individual instances but also generates sharper segmentation masks.

**Figure 8:**
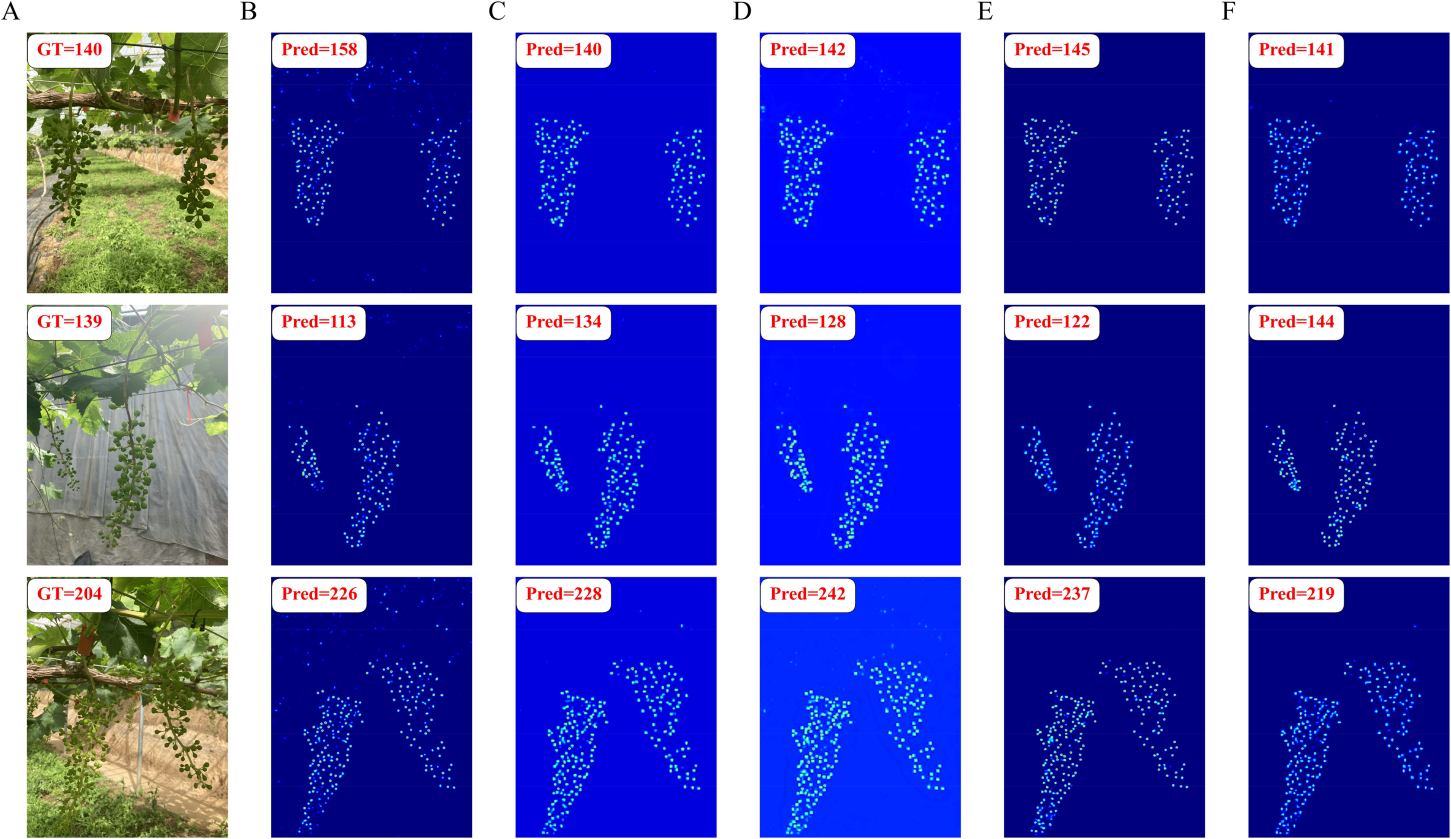
Visualization of density map regression results with different models. **(A)** Original image, **(B)** MCNN, **(C)** CSRNet, **(D)** CANNet, **(E)** SCAR, and **(F)** MVDNet.

**Figure 9:**
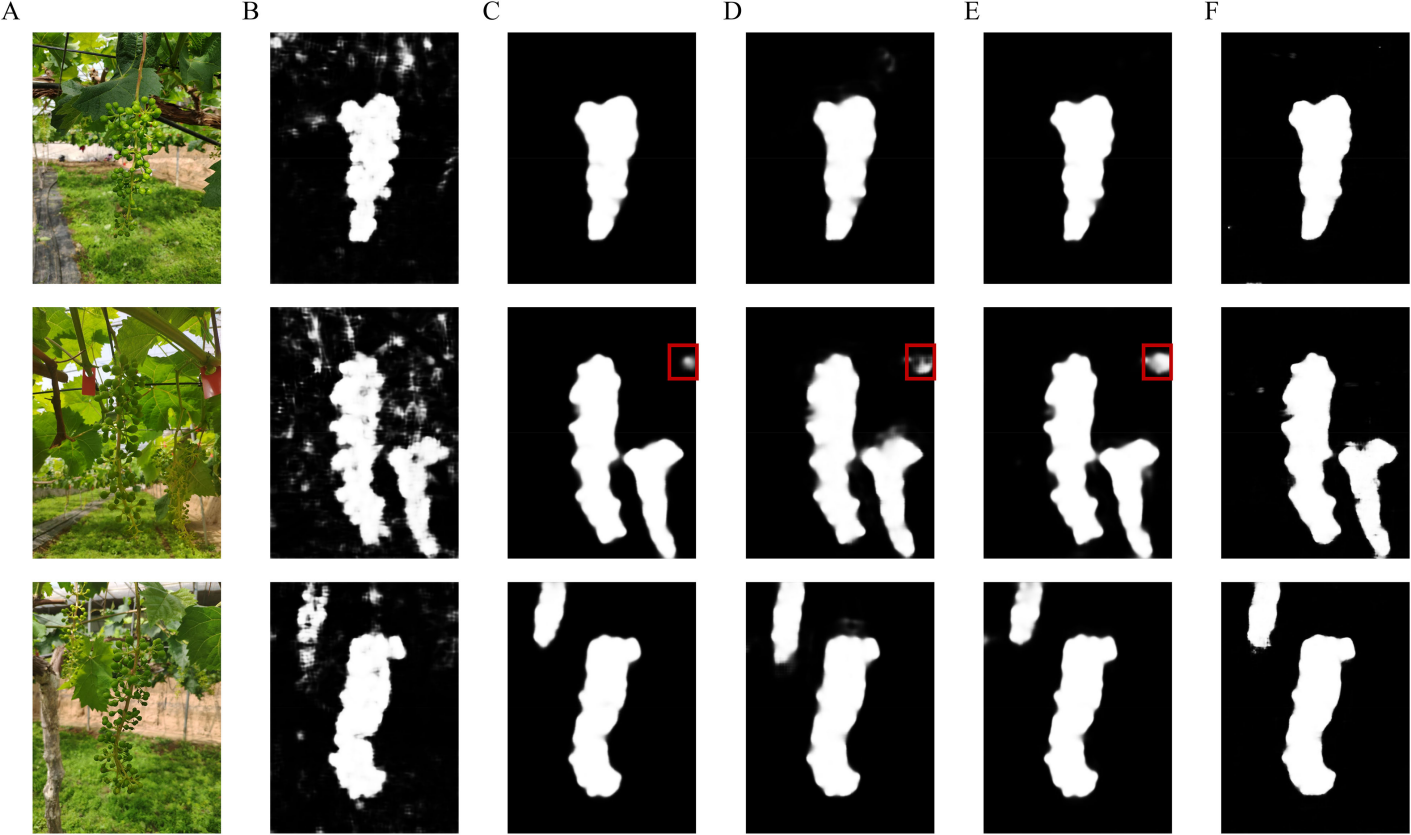
Visualization results of segmentation with different models. **(A)** Original image, **(B)** MCNN, **(C)** CSRNet, **(D)** CANNet, **(E)** SCAR, and **(F)** MVDNet.

To provide a more intuitive demonstration of the density map regression and segmentation performance with all models, the visualization results for each model were shown in **Figures 8** and **9**. In the density map regression results, MCNN exhibited relatively poor performance, misclassifying background foliage as grape fruits and including them in the statistics. The other models exhibited varying degrees of counting accuracy, with MVDNet demonstrating regression precision closest to the ground truth and exhibiting greater stability. This indicates that MVDNet could more accurately localize and regress fruit regions, effectively suppressing interference from complex backgrounds such as foliage and shadows. Particularly in areas of dense fruit distribution, this model maintained good detail resolution, avoiding excessive overlap or missed detection of adjacent fruits.

For the segmentation results of grape clusters, MCNN produced relatively coarse segmentation, while other models demonstrated comparatively superior segmentation performance. MVDNet yielded clearer boundaries and finer segmentation results, primarily due to its adoption of more advanced feature fusion mechanisms and decoder architecture. Its encoder extracted multi-scale, high-semantic-level features, while the decoder effectively merges detailed information (such as edges and textures) from shallow layers with semantic information from deep layers through progressive upsampling and skip connections. This design enabled the model to grasp the overall shape structure while precisely restoring subtle edge variations during target contour reconstruction, thereby generating segmentation results with clear boundaries and complete internal filling.

For in-depth analysis of the interactive effects between counting and segmentation tasks, the counting task branch and segmentation task branch were trained separately. The results were presented in **Table 6**. It was clearly observable that under the dual-task model, the MAE (7.7) and RMSE (12.6) of the counting task were identical to those of the independently trained counting task model. Simultaneously, the MIoU (0.90) of the segmentation task achieved the same performance as the standalone segmentation model. This outcome demonstrated that the proposed dual-task architecture achieves parallel multi-task learning whilst preserving the independent performance of each task, without exhibiting significant cross-task interference or gradient competition issues.

**Table 6:** Evaluation results for independent-task and dual-task models.

Task	MAE	RMSE	MIoU
Counting task	7.7	12.6	_
Segmentation task	_	_	0.90
Dual-task (our method)	7.7	12.6	0.90

With the purpose of analyzing the parameters sensitivity of DBSCAN, we conducted experiments by setting ϵ to 10, 15, and 20, and minPts to 1, 2, and 3, respectively. The results were shown in **Table 7**. The parameter minPts critically affected the results. When minPts=1, instance segmentation achieved optimal performance, while *ϵ* had no impact within the tested range. In the MVDNet, the segmentation task branch already provided high-quality masks, and DBSCAN was only responsible for clustering the pixels within the masks to distinguish different grape bunches. The parameter minPts=1 performed best, indicating that there might be fine gaps or adhesions inside the grape cluster masks, and a low-density requirement could more actively connect pixels belonging to the same cluster into a cohesive entity, avoiding under-segmentation. Meanwhile, the parameter ϵ had no effect within the tested range (10-20), indicating that this threshold was sufficient to cover the maximum pixel distance within a single cluster, and further increasing it did not help improve the clustering results.

**Table 7:** Parameter sensitivity analysis of DBSCAN.

ϵ	minPts	mIoU
10	1	0.84
10	2	0.78
10	3	0.78
15	1	0.84
15	2	0.78
15	3	0.78
20	1	0.84
20	2	0.78
20	3	0.78

**Figure 10:**
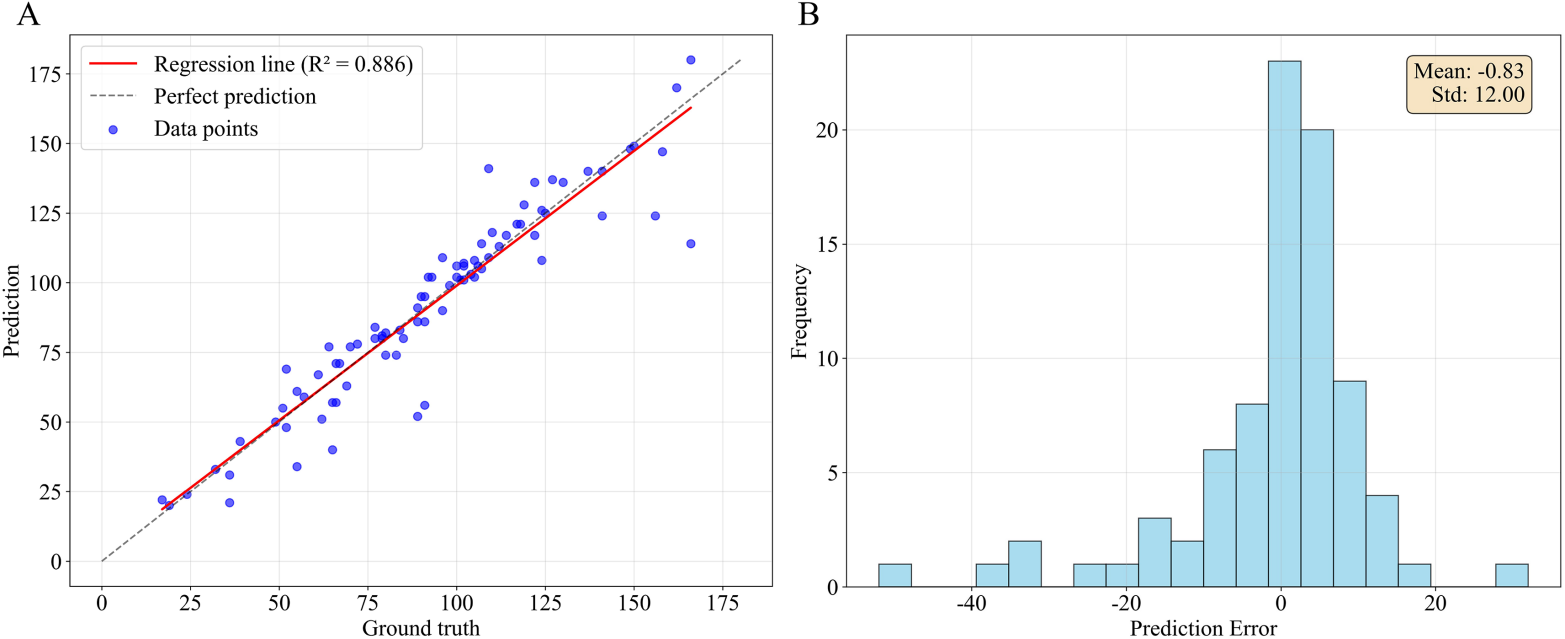
Results of grape berry counting per cluster. **(A)** Regression analysis of berry counting per bunch, and **(B)** Distribution of prediction errors.

To evaluate the performance of our method in counting berries on individual grape clusters, a regression analysis on the predicted berry counts within the test dataset was conducted. By matching predicted grape cluster instances with annotated cluster masks, the predicted berry count for a given cluster was aligned with its actual berry count. This approach guarantees the validity of the regression analysis for individual clusters. The *R*^2^ (see Equation 16) was used to evaluate the precision of our method, as shown in **Figure 10**.

(16) $$R^{2}=1-\frac{\sum {\left(B_{i}^{\text{GT}}-B_{i}^{\text{Pred}}\right)}^{2}}{\sum {\left(B_{i}^{\text{GT}}-\bar{B}\right)}^{2}}$$

where $B_{i}^{\text{GT}}$ is the true number of berries per cluster, $B_{i}^{\text{Pred}}$ is the predicted number of berries per cluster, $\bar{B}$ is the average value of $B_{i}^{\text{GT}}$.

The regression analysis demonstrated a strong linear relationship between the predicted and ground-truth values, with an *R*² of 0.886, reflecting a high level of predictive accuracy. The distribution of prediction errors showed a mean error of –0.83 and a standard deviation of 12.00, suggesting that this method exhibit a slight underestimation bias, though the overall error distribution appears approximately symmetric around zero. The majority of errors were concentrated within a reasonable range, with few outliers beyond ±20. These results indicated that the method is robust and reliable for estimating grape berry numbers per cluster under the evaluated conditions.

**Figure 11:**
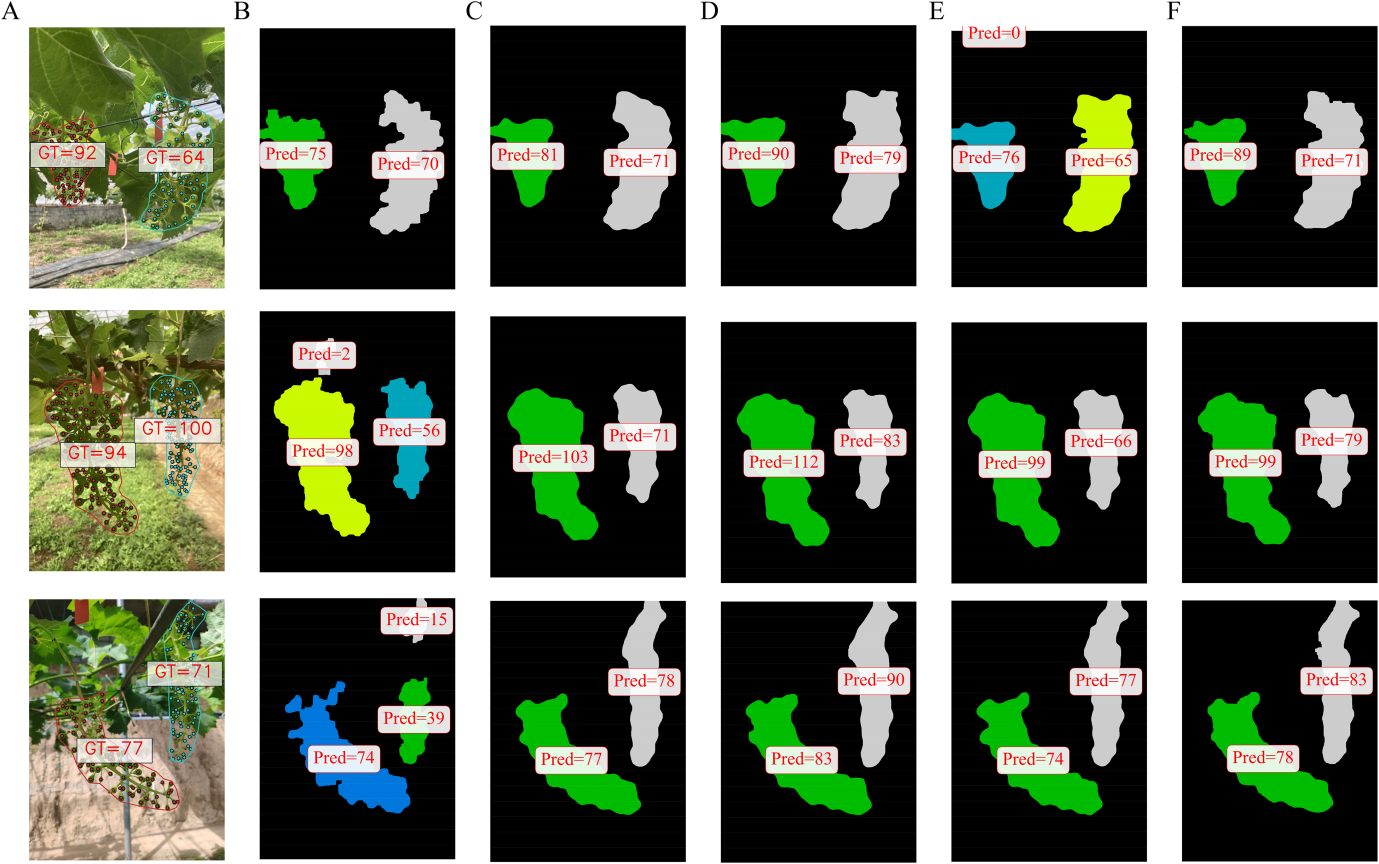
Visualization of berry counting for per cluster with different models. **(A)** Original image, **(B)** MCNN, **(C)** CSRNet, **(D)** CANNet, **(E)** SCAR, and **(F)** MVDNet.

To further validate the performance of our method in counting berries per grape cluster, the visualization results from different models with the same post-processing were compared. As shown in **Figure 11**, MVDNet demonstrated clear advantages in accurately estimating the number of grape berries per cluster. In contrast to MCNN, which tended to produce blurred or overly diffuse density responses in high-density berry regions, MVDNet provided more localized and precise density estimates, effectively minimizing background interference. When compared to other models, MVDNet maintained better spatial consistency and granularity in dense areas, reducing both over-counting in ambiguous regions and under-counting in occluded areas. The visual results suggested that MVDNet achieve superior structural awareness and berry-level discriminability, leading to more reliable and fine-grained counting performance in complex vineyard environments.

## 4 Discussion

Cluster-level berry counting represents a more complex task than berry-level counting, as it requires not only the identification of individual berries but also the accurate assignment of these berries to their respective clusters for precise per-cluster estimation. While recent studies have made significant strides in addressing this challenge, several methodological limitations persist.

Several deep learning frameworks have been developed for cluster-level berry counting with integrated functionality. Woo et al. (2023) proposed a method combining object detection and a random forest regressor, though its generalization capability may be limited by dependency on handcrafted bounding-box features. Yang et al. (2024) introduced the PMGDC framework using a U-Net architecture, yet its multi-step post-processing incurs computational overhead and sensitivity to severe occlusions. Another approach, Mask-GK, Yang et al. (2025) employs mask-based Gaussian kernels for joint segmentation and counting but requires labor-intensive instance-level annotations. Furthermore, this method may struggle with accurately segmenting large or highly irregular berry clusters.

Our approach is compared with existing methods as shown in the **Table 8**. Despite our custom dataset being relatively small (only 779 images) and featuring high image resolution (1,024 × 1,365), we achieved a MAE of 7.7 by combining MVDNet and DBSCAN techniques. This result outperforms the 9.32 MAE obtained by Yang et al. (2025) on larger image dimensions and mature grapes; simultaneously, our approach achieves an acceptable accuracy level despite limited data volume, as evidenced by its MAE of 7.7 compared to the 2.60 MAE attained by Woo et al. (2023) using a large-scale dataset (26,230 images) with YOLOv5s and a Random Forest Regressor. However, this method achieves exceptional accuracy as only one cluster of grapes is present in the entire image during each berry count. Moreover, although Yang et al. (2024) conducted evaluations across multiple growth stages, their assessment metrics, MRD and 1-FVU, differ from the MAE employed in this method, rendering direct comparison challenging. Overall, this approach achieves competitive performance even under conditions of limited data volume and large image dimensions, demonstrating sound practical potential and scalability.

**Table 8:** Comparison of different methods.

Paper reference	Dataset	Grape growth stage	Image size	Method	Berry-per-cluster counting performance (MAE, berries)
Woo et al. (2023)	Custom dataset (26,230 images);	Berry thinning season	640 x 640	YOLOv5s + Random Forest Regressor	2.60
Yang et al. (2024)	WGISD (300 images) + Chengdu (100 images) + BpGC (137 images)	Multiple growth stages	1,280 x 960	PMGDC	MRD: 0.142, 1-FVU: 0.865
Yang et al. (2025)	GBISC dataset (150 images)	Mature stage	2048 x 1536	Mask-GK	9.32 (whole image)
Our method	Custom dataset (779 images)	Berry thinning season	1,024 x 1,365	MVDNet + DBSCAN	7.7

In this context, our approach offers a promising alternative by striking a balance between accuracy, efficiency, and robustness. The architectural design of MVDNet, featuring a dual-branch structure for simultaneous density map regression and bunch segmentation, coupled with multi-scale feature fusion and an efficient attention mechanism, enables robust feature learning from complex imagery. The subsequent post-processing algorithm effectively translates these features into accurate per-cluster berry counts, as evidenced by the high *R*² value on the test set. The low parameter count of our model further underscores its suitability for deployment on resource-constrained edge devices, facilitating practical in-field applications such as guided berry thinning.

**Figure 12:**
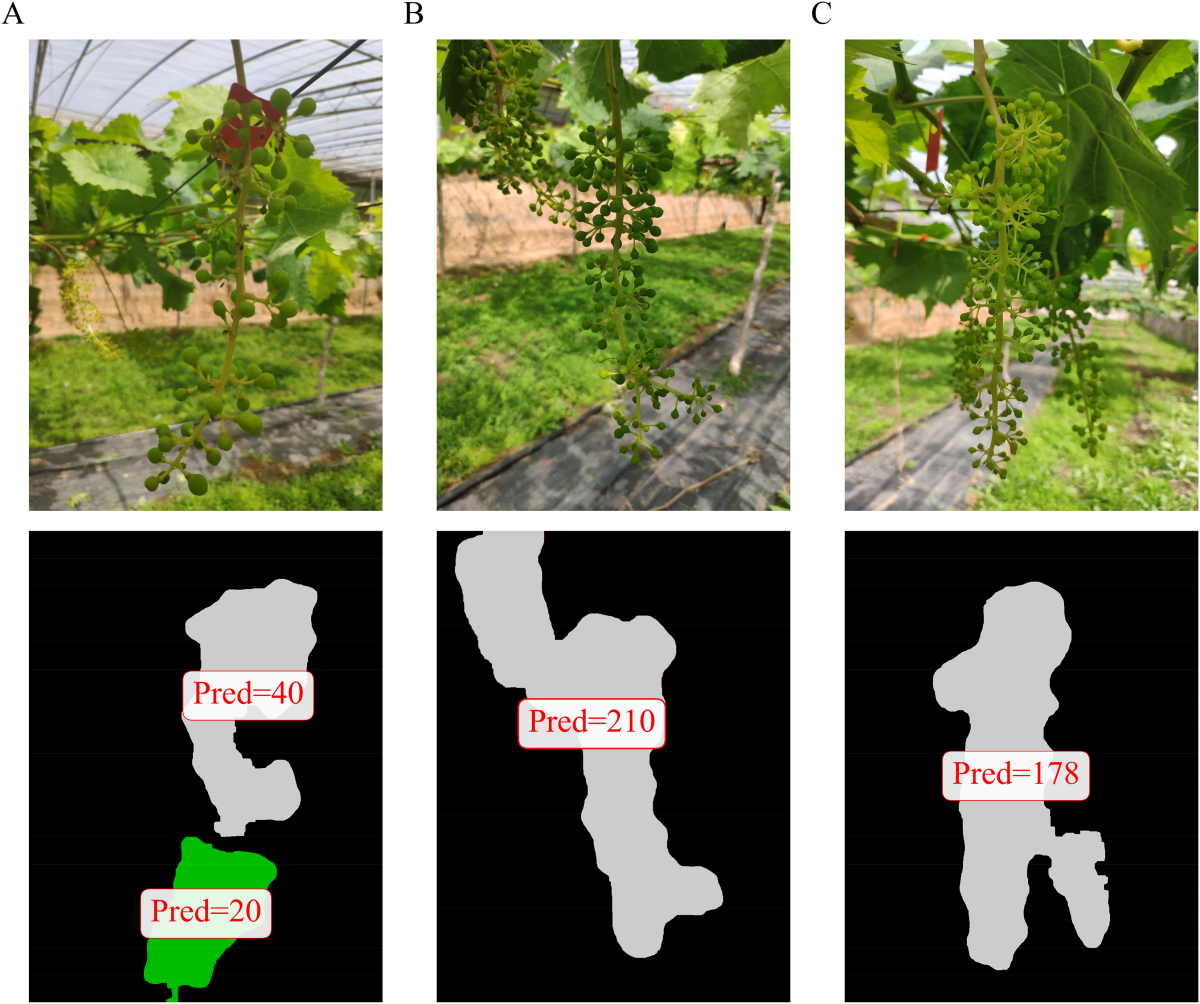
Counting failure cases. **(A)** Case 1 **(B)** Case 2 **(C)** Case 3.

Despite the promising results, this study has certain limitations. First, the accuracy of the final per-cluster berry count is contingent upon the performance of both the bunch segmentation branch and the density map regression branch; in cases of extremely dense and overlapping clusters, segmentation inaccuracies may propagate to the counting stage, and the density estimation model may also struggle to accurately capture complex occlusion and perspective distortions. As shown in **Figure 12A**, since the bunch was too loose, MVDNet identified it as two separate bunches. **Figures 12B**, **C** showed two grape bunches adhering to one another, which were identified as a single cluster during segmentation. Second, the model was trained and validated on a specific dataset, and its generalizability across dramatically different grape varieties, training systems, or lighting conditions remains to be further investigated.

Based on the limitations, future work will first focus on improving model robustness by exploring advanced architectures like Vision Transformers and deeper multi-branch fusion to better handle occlusions. Secondly, incorporating 3D data and synergistic multi-task learning, such as joint center-point detection, will be investigated to improve accuracy in dense clusters. To address generalizability, a primary goal is building a large-scale, diverse dataset spanning multiple varieties and growth conditions. Concurrently, we will research domain adaptation and few-shot learning techniques to minimize data needs for new environments. For practical deployment, efforts will be made to develop lightweight models for edge computing on mobile devices.

## 5 Conclusion

In this study, a precise in-cluster grape berry counting method to guide thinning operations is proposed. The presented method comprises two integral components: a dual-branch network that integrates density map regression and bunch segmentation, named MVDNet, and a subsequent post-processing algorithm. The MVDNet features an efficient architecture: the Front-end employs a UIB structure for powerful feature extraction, while the Back-end adopts multi-scale feature fusion to carefully reconstruct spatial details and improve robustness against occlusion and scale variation. Additionally, the model incorporates the parameter-free SimAM attention mechanism, which effectively enhances salient berry features while suppressing interference from complex backgrounds. Extensive experimental validation demonstrates the outstanding performance of MVDNet, achieving an MAE of 7.7, RMSE of 12.6, and MIoU of 0.90 on the test dataset. Meanwhile, the model maintains high accuracy with very low computational cost—containing only 3.372M parameters, which is significantly fewer than comparable high-performance models. Moreover, MVDNet exhibits superior robustness in addressing inherent challenges of in-field berry counting, including high berry density, severe occlusion, large intra-cluster scale variation, and complex natural backgrounds.

Building upon the output of MVDNet, a post-processing algorithm based on image processing to determine the number of grapes per cluster is proposed. Regression analysis on the test set for per-bunch berry count yielded an *R*² of 0.886, with a mean error of -0.83 and a standard deviation of 12, indicating high prediction accuracy. Overall, our method proves to be a highly accurate, efficient, and practical solution, well-suited for deployment on resource-constrained edge devices. Its development provides viticulturists with a powerful automated tool to support the critical and labor-intensive task of berry thinning, enabling more precise yield management and contributing to enhanced grape quality and vineyard productivity.

## Data Availability

The data analyzed in this study is subject to the following licenses/restrictions: The data presented in this study are available on request from the corresponding author. Requests to access these datasets should be directed to duyp@sdau.edu.cn.
